# Local steroid injection in severe idiopathic granulomatous mastitis as a new first-line treatment modality with promising therapeutic efficacy

**DOI:** 10.3389/fmed.2023.1251851

**Published:** 2023-10-04

**Authors:** Neslihan Cabioglu, Cihan Uras, Halime Mutlu, Derya Sezgin, Selman Emiroglu, Onur Dulgeroglu, Ravza Yilmaz, Mustafa Tukenmez, Akif Enes Arikan, Halil Kara, Mahmut Muslumanoglu

**Affiliations:** ^1^Department of Surgery, Istanbul Faculty of Medicine, Istanbul University, Istanbul, Türkiye; ^2^Department of Surgery, School of Medicine, Acibadem University, Istanbul, Türkiye; ^3^Department of Radiology, Istanbul Faculty of Medicine, Istanbul University, Istanbul, Türkiye

**Keywords:** idiopathic granulomatous mastitis, local steroid injection, intralesional steroid injection, systemic steroid use, topical steroid-containing pomades

## Abstract

**Background:**

Intralesional steroid injection has recently evolved as a novel treatment modality for localized idiopathic granulomatous mastitis (= IGM). We aimed to explore the therapeutic efficacy of local steroid injections (LSI) in patients with severe IGM.

**Methods:**

Fifty-one patients diagnosed with severe IGM were included in the study and treated with either local steroid injection (LSI) alone (*n* = 25) or combined LSI with systemic oral steroid treatment (OST) (*n* = 26). The local steroid injection protocol included an intralesional triamcinolone acetonide injection into the palpable granulomas every 4-week, and topical administration of steroid-containing pomades twice a day on the affected surface of the breast. Patients with a combined LSI and OST received low-dose oral methylprednisolone (<16 mg).

**Results:**

Patients with LSI alone required more LSI applications than those in the combined LSI with OST group (LSI: 5 ± 2.9; vs. LSI/OST: 3.5 ± 2.5; *p* = 0.080) to obtain an effective optimum therapeutic response. At a median of 12 months (range, 4–42), no difference was found in complete response rates between patients in the LSI group and the combined LSI group with OST (52 vs. 53.9%, *p* = 0.999). However, steroid-related systemic side effects were lower in the LSI alone group (*p* < 0.008).

**Conclusion:**

Local steroid injection could be considered as the first-line treatment in patients with severe IGM until a therapeutic response has been obtained either as the sole treatment modality or combined with oral steroids. Compared with systemic oral steroid therapy, local steroid administration can be considered a new treatment modality with fewer side effects.

## Introduction

Idiopathic granulomatous mastitis (IGM) was first reported by Kessler and Wolloch in 1972 (1). IGM is an uncommon chronic inflammatory condition of the breast with an unclear etiology that may be associated with immune dysfunction, such as autoimmunity (2–6). Cases of IGM have been mostly reported in various Asian countries, including Türkiye, suggesting that environmental and genetic factors may also play an important role in the underlying etiology of the disease (7). Most patients were women of childbearing age with a history of pregnancy or lactation. IGM is histologically characterized by necrotizing chronic granulomatous lobulitis and abscess formation. IGM mostly presents with nipple distortion, breast masses, and skin erythema. IGM has therefore been an important entity in breast diseases that should be pathologically distinguished from malignant lesions at initial presentation because it can clinically mimic breast cancer (1, 8). However, IGM is not a known cause of cancer.

There is no consensus regarding the therapeutic management of IGM. The most commonly used medical treatment is corticosteroids, which were first proposed by DeHertogh et al. in 1980 as systemic oral administration (9). Systemic use of corticosteroids has played a major role in the treatment of IGM either alone or combined with surgery so far (10–13). However, owing to the remarkable side effects of corticosteroids, topical corticosteroid administration has been the backstone in the management of IGM as the sole treatment modality or along with systemic steroid administration (14–17).

Intralesional steroid injection has evolved as a novel treatment modality in recent years (18–24). Recent studies have focused on intralesional steroid injection alone compared with other modalities, including oral steroid treatment (OST) alone or surgical therapy in cases with localized IGM. In this study, we aimed to explore the therapeutic efficacy and adverse effect profile of local steroid injection (LSI) alone compared with the combined therapy of LSI and low-dose steroid therapy in severe IGM.

## Materials and methods

Between June 2019 and June 2022, fifty-one patients from surgical clinics of two centers, Istanbul University, Istanbul Faculty of Medicine, and Acibadem University, School of Medicine, were included in the prospective registry study. This study was approved by the Ethical Committee of the Istanbul University, Istanbul Faculty of Medicine. Informed consent was obtained from all the patients before the procedure. The patients’ demographic features, complaints, imaging methods at diagnosis, pathology results, treatment method, and duration of treatment were recorded. The prospectively maintained data were analyzed in terms of clinical characteristics, therapy response and side effects in patients included into the study.

**FIGURE 1:**
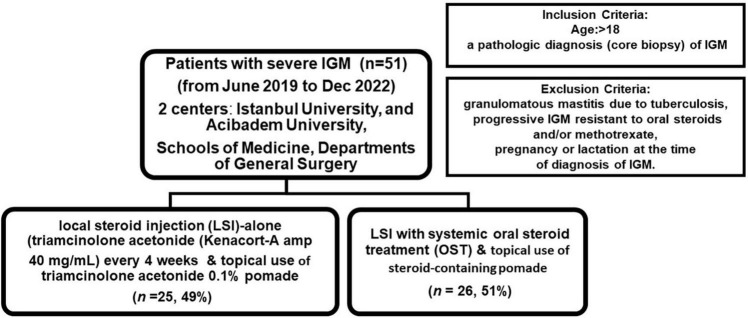
Progressive granulomatous mastitis resistant to oral steroids.

The inclusion and exclusion criteria and the study design are shown in Figure 1. All patients aged >18 years with a pathological diagnosis of granulomatous mastitis were included in the prospective registry study. All patients underwent diagnostic work-up by breast ultrasound, whereas breast magnetic resonance imaging (MRI) was performed at the discretion of the surgeon (*n* = 18, 31.4%). Of those >35 years (*n* = 14), 78.6% (*n* = 11) also had a mammogram (MMG) at the initial diagnosis to exclude malignancy. A core needle biopsy performed under ultrasound revealed multinucleated Langhans-type giant cells, lymphocytes, polymorphonuclear leukocytes, and plasma cells infiltrating the breast lobules, indicating chronic lobulitis forming non-caseating granulomas.

To define IGM, breast tuberculosis was excluded by the Quantiferon^®^-TB Gold test and/or PPD skin test at the initial diagnosis of patients. Furthermore, microbiological cultures and/or reverse transcriptase-polymerase chain reaction (RT-PCR) tests remained sterile and were obtained from pus samples of patients presenting with breast abscess, open wound, and/or discharge due to cutaneous fistulae. Blood tests were also ordered to investigate the ds-DNA, anti-nuclear antibodies, and rheumatoid factor to exclude rheumatologic diseases, such as systemic lupus erythematosus and rheumatoid arthritis. Patients with concurrent bilateral disease and severe systemic signs, such as disseminated erythema nodosum and/or arthritis, were excluded from the study and were treated with a standard steroid regimen. Other exclusion criteria were a diagnosis of progressive granulomatous mastitis resistant to oral steroids and/or methotrexate and the presence of pregnancy or lactation at the time of diagnosis of IGM.

All patients in this prospective registry had clinically severe IGM characteristics, presenting with painful granulomas with either abscess or cutaneous fistula formation with or without discharge, erythema, and mass according to the classification criteria that was recently described in an international multidisciplinary consensus (25).

### Treatment protocol

**FIGURE 2:**
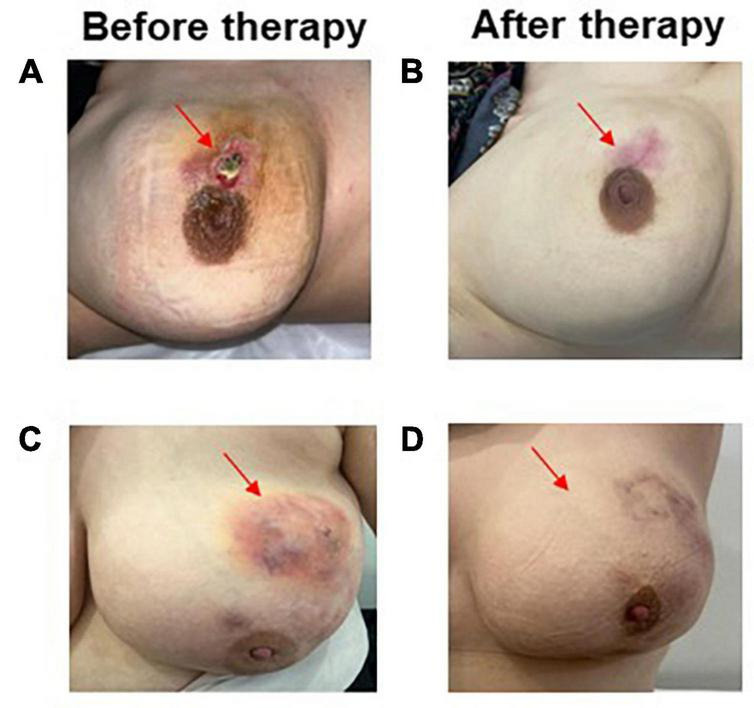
Patient response before **(A)** and after the LSI alone **(B)** disappearance of the patient’s IGM lesion as open wound formation following 4 LSI applications. Complete response in a patient treated with LSI with OST showing disappearance of abscess formation **(C,D)**.

Patients were treated with either local steroid injection (LSI) alone (*n* = 25, 49%) or combined LSI and systemic OST (*n* = 26, 51%). The local steroid injection protocol included intralesional triamcinolone acetonide (Kenacort-A amp. 40 mg/mL; Deva, Istanbul, Turkey) injection into palpable granulomas every 4-weeks (Figure 2a), and topical administration of triamcinolone acetonide 0.1% pomade (Kenocort-A 0.1%, 20-g pomade, Deva, Istanbul, Turkey) on the skin of the granulomas in the affected breast twice a day until an optimum therapeutic response has been obtained. The active ingredient was diluted with saline solution at a ratio of 1/10, and the solution was injected with a syringe (22 G) into the granuloma and surrounding inflammatory tissues around the abscess and fistula tract in the presence of abscess or fistula. Patients with multiple active lesions having diffuse disease, a maximum of 2 index lesions were injected separately at different locations with a total dose of 40 mg/10 cc if those lesions were >5 cm away from each other, as described previously (19). Patients in the LSI combined with OST group received low-dose oral methylprednisolone starting mostly at a low dose of 4 or 8 mg (<16 mg) per day, depending on the severity of the IGM, presence of systemic symptoms including erythema nodosum, and at the discretion of the surgeon in the present study.

**FIGURE 3:**
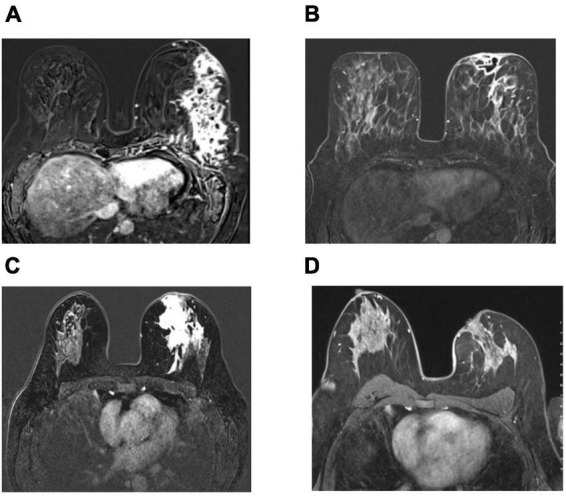
Axial pretreatment contrast enhanced images **(A,C)** show contrast enhancement at the inflamed area extending to the prepektoral region with the collection area that opened to the skin **(A)**, and contrast enhancement at the inflamed area extending to the periareolar region **(C)**. Complete radiological response after therapy in 2 patients treated with local steroid injection and oral low-dose steroid use as complete disappearance of contrast enhancement in subtraction images in MRI **(B,D)**.

Response to therapy was determined by physical examination and/or imaging findings before and after therapy, as described in Table 1 and shown in Figures 2, 3. Briefly, a partial response and minimal response were defined as the disappearance of clinical symptoms and inflammatory lesions ≥50% and <50%, respectively, following treatment. Similarly, complete clinical response was defined as the complete disappearance of clinical symptoms and complaints. Recurrence was defined as a relapse of symptoms after treatment completion. Outcomes were evaluated based on any recurrence in the affected breast at 1 year as determined according to the median follow-up time of each cohort.

**TABLE 1:** Classification of the clinical response to therapy.

Clinical response	By physical exam and radiology (ultrasound or magnetic resonance imaging before and after therapy)
Complete response	Complete disappearance of complaints with clinical recovery including pain and discharge from fistulas and inflammatory signs in the breast including the closure of fistula orifices and/or skin erosions
Partial response	The disappearance of clinical symptoms and inflammatory lesions ≥50% following treatment
Minimal response	The disappearance of clinical symptoms and inflammatory lesions <50% following treatment
No response	No improvement in symptoms and radiological findings
Progression	Worsening of clinical symptoms and radiological findings

### Statistical analysis

SPSS 25 software (Statistical Package for Social Sciences; SPSS, IBM Corp., Armonk, NY, USA) was used for the statistical analyses. Continuous variables including age, size of the granulomas, number of LSI-applications, steroid dose, and time of steroid use were analyzed using the Mann-Whitney *U*-test to compare the differences between the two treatment groups. In cases of multiple granulomas, the sum of the sizes was considered when evaluating the severity of the disease. Categorical variables, including the frequencies of clinical or treatment characteristics, response to therapy, 1-year recurrence, and complication rates, were evaluated using the Pearson Chi-square or Fisher’s exact tests to estimate the differences between the LSI-alone and the LSI with OST groups. Fisher’s exact test was used when the expected values in any of the cells of a contingency table are below 5, whereas Pearson Chi-square test was utilized when the expected values ≥5. A *p*-value equal to or less than 0.05 was considered statistical significance.

## Results

Patient demographic characteristics, physical examination findings, and treatment characteristics are presented in Tables 2, 3, respectively. The median age of the patients was 37 years (range, 24–52). Almost all the patients were premenopausal (98%) and had at least one live birth (100%). The median age of the first live births was 24 (range, 17–36), and the median number of live births was 2 (range, 1–4). The oral contraceptive use rate was also very low, at 3.9% in the entire cohort.

**TABLE 2:** Demographic and clinical characteristics of patients with idiopathic granulomatous mastitis treated with local steroid injections with/without oral steroids.

Patient characteristics	Total (*N* = 51)	Local steroid injection only (*n* = 25)	Local steroid injection with oral steroid treatment (*n* = 26)	*p-value*
Median age (range, min- max)	37 (range, 24–52)	37 (range, 24–47)	36 (range, 25–52)	0.792^a^
Menopausal status: premenopausal	98% (50/51)	100% (25/25)	96.2% (25/26)	0.999^b^
Nulliparous	0% (0/51)	0% (0/25)	0% (0/26)	0.999^b^
Median age of first live birth (range, min-max)	24 (17–36)	26 (17–32)	22 (18–36)	0.080^a^
Median number of live births	2 (1–4)	2 (1–4)	2 (1–4)	0.072^a^
Oral contraceptive use (+)	3.9% (2/51)	0% (0/25)	7.7% (2/26)	0.480^b^
**Physical exam findings:**				
Mass	100% (51/51)	100% (25/25)	100% (26/26)	0.999^b^
Pain	98% (50/51)	100% (25/25)	96.2% (25/26)	0.491^b^
Erythema or abscess formation	94.1% (49/51)	100% (25/25)	88.5% (24/26)	0.480^b^
Cutaneous fistula formation	51% (26/51)	52% (13/25)	50% (13/26)	0.999^c^
Open wound formation	37.2% (19/51)	40% (10/25)	34.6% (9/26)	0.691^c^
Multifocality/multicentricity	37.3% (19/51)	28% (7/25)	50% (13/26)	0.108^c^
The median size of the granuloma [(mm) ± SD/range]	40 ± 18.4 mm (range, 20–90)	50 ± 15.9 mm (range, 20–80 mm)	36.5 ± 19.6 mm (range, 20–90 mm)	0.115^a^
Bilaterality	5.9% (3/51)	8% (2/25)	3.8% (1/26)	0.610^b^
Erythema nodosum	9.8% (5/51)	0% (0/25)	19.2% (5/26)	0.051^b^

^a^Mann-Whitney U, ^b^Fisher’s exact test, or ^c^Pearson Chi-square test were used in statistical analyses.

**TABLE 3:** Treatment and response characteristics and side effects of patients with idiopathic granulomatous mastitis treated with local steroid injections with/without oral steroids.

Patient characteristics	Total (*N* = 51)	Local steroid injection only (*n* = 25)	Local steroid injection with oral steroid treatment (*n* = 26)	*p*-value
Median follow-up time (months)	12 (range, 4–42)	8 (range, 4–42)	13.5 (range, 4–28)	0.248^a^
Number of LSI applications (Median ± SD)	4 ± 2.8	5 ± 2.9	3.5 ± 2.5	0.080^a^
Number of LSI applications:				0.069^b^
<4	37.3% (19/51)	24% (6/25)	50% (13/26)	
4–7	45.1% (23/51)	48% (12/25)	42.3% (11/26)	
>7	17.6% (9/51)	28% (7/25)	7.7% (2/26)	
>7LSI applications	17.6% (9/51)	28% (7/25)	7.7% (2/26)	0.075^b^
Clinical therapy response pattern*:				0.544^b^
Complete response	53% (27/51)	52% (13/25)	53.9% (14/26)	
Partial response	39.2% (20/51)	44% (11/25)	34.6% (9/26)	
Minimal response	7.8% (4/51)	4% (1/25)	11.5% (3/26)	
Median week of steroid use	N.A.	N.A.	8 (range, 2–48)	N.A.
Median maximum methyl prednisolone dose per day	N.A.	N.A.	4 mg (range, 4–16 mg)	N.A.
1-year recurrence	9.8% (5/51)	4% (1/25)	15.4% (4/26)	0.350^b^
**Any systemic complications due to the steroids	51% (26/51)	32% (8/25)	69.2% (18/26)	0.008^c^

^a^Mann-Whitney U, ^b^Fisher’s exact test, or ^c^Pearson Chi-square test were used in statistical analyses. N.A., not applicable.

*Clinical therapy response has been shown in Table 1.

**Systemic side effects: weight gain, face edema, menstrual irregularity, hirsutism, acne, hair loss, weakness, hypertension, and skin thinning.

Patients in this cohort had severe symptoms of IGM, such as mass (100%), redness or abscess formation (94.1%), painful granulomas (98%), fistula formation (51%), open wound formation (37.2%), and multifocal/multicentric granulomas (37.3%) (Table 2). The median size of the granulomas was 40 ± 18.4 mm (range, 20–90). Few patients had bilateral disease (5.9%), which was not concurrent, and localized erythema nodosum (9.8%), which disappeared very rapidly with the initiation of therapy. All patients with erythema nodosum were treated with oral steroids combined with LSI. No significant differences were found in the demographic and clinical physical examination findings between patients in the LSI-alone and LSI with OST groups, except for erythema nodosum that did not reach the statistical significance (Table 2).

The median number of LSI applications was 4 ± 2.8 in the whole cohort. Patients with LSI alone were more likely to have LSIs > 7 than those in the combined treatment group (LSI: 28% vs. LSI/OST: 7.7%; *p* = 0.075) to obtain an effective therapeutic response that did not reach the statistical significance. Patients in the LSI with OST group received oral steroids at a median dose of 4 mg (range, 4–16 mg) for a median of 8 weeks (range, 2–48) along with the LSIs. Of the 26 patients with OST, the majority (80.8%, *n* = 21) received oral steroids at a maximum dose of 4 or 8 mg/day, whereas the remaining few patients received a dose of 16 mg/day. Cortisone therapy was discontinued gradually after the second LSI.

At a median of 12 months (range, 4–42), significant clinical resolution was demonstrated in all patients as assessed by physical examination and imaging, including MRI, in some patients, as shown in Figures 2, 3. More than half of the patients (53%) showed a complete clinical response with the disappearance of granulomas and clinical signs associated with IGM following therapy in the whole cohort. Of the remaining patients, the majority had partial responses (39.2%), whereas a few (7.8%) showed a minimal response. No significant differences were found in response patterns between the two treatment groups. Interestingly, patients in the LSI alone (52%) and LSI with OST (53.9%) groups showed almost equal complete response rates. However, patients in the LSI-alone group required a longer duration of LSI therapy to achieve an optimal therapeutic response. Similarly, no significant difference was found in the 1-year recurrence rates between the two treatment groups (LSI, 4% vs. LSI with OST, 15.4%; *p* = 0.350) (Table 3). One patient (3.8%) in the combined therapy group with a partial response underwent local excision of the lesion following the completion of the seventh LSI application.

The most common steroid-related side effects were weight gain (29.4%), hirsutism (27.5%), facial edema (21.6%), menstrual irregularities (19.2%), hair loss (11.8%), skin thinning (11.8%), and weakness (11.8%; Table 4). As expected, presence of any steroid-related systemic side effects were lower in the LSI alone group (8/25, 32%) than in the LSI with OST group (18/26, 69.2%) (*p* = 0.008). Of note, patients with LSI alone were less likely to gain weight following therapy than those in the combined group. Although a decreasing trend was observed for other side effects, these differences did not reach statistical significance (Table 4). Furthermore, as local side effects, only one patient of 51 patients (1.9%) had infection at the injection area that appeared within 48 h following LSI application and resolved rapidly with the use of oral antibiotics.

**TABLE 4:** Side effects of patients with idiopathic granulomatous mastitis treated with local steroid injections with/without oral steroids.

Systemic effects	Total (*N* = 51)	Local steroid injection only (*n* = 25)	Local steroid injection with oral steroid treatment (*n* = 26)	*p*-value
Weight gain	29.4% (15/51)	16% (4/25)	42.3% (11/26)	0.039^a^
Face edema	21.6% (11/51)	16% (4/25)	26.9% (7/26)	0.343^a^
Hirsutism	27.5% (14/51)	24% (6/25)	30.8% (8/26)	0.588^a^
Acne	5.9% (3/51)	4% (1/25)	7.7% (2/26)	0.999^b^
Hair loss	11.8% (6/51)	12% (3/25)	11.5% (3/26)	0.999^b^
Skin thinning	11.8% (6/51)	4% (1/25)	19.2% (5/26)	0.191^b^
Menstrual irregularity	13.7% (7/51)	4% (2/25)	19.2% (5/26)	0.419^b^
weakness	11.8% (6/51)	4% (1/25)	19.2% (5/26)	0.191^b^
Hypertension	3.9% (2/51)	0% (0/25)	7.7% (2/26)	0.490^b^

^a^Pearson Chi-square, or. ^b^Fisher’s exact test were used in statistical analyses.

## Discussion

Idiopathic granulomatous mastitis is a chronic, benign inflammatory breast disease with an uncertain etiology. Immunological dysfunction has been claimed in the underlying pathogenesis to trigger an inflammatory autoimmune response in its own breast epithelial tissue, causing chronic lobulitis as non-caseating granulomas (2–7). Despite the lack of consensus on the optimal treatment modality, two treatment options exist: administration of immunosuppressive agents including steroids, and surgical excision, mainly in cases with localized IGM (8–13, 26). Owing to the undesirable side effects of systemic oral use of steroids, topical steroid applications, including steroid-containing pomades, have commonly been used as the sole treatment modality in patients with mild IGM or in combination with systemic low-dose steroids in more severe cases in the contemporary management of IGM (14, 15).

In medical practice, steroids are applied locally to treat various inflammatory or non-inflammatory conditions, particularly in orthopedic and rheumatological disorders (27). Hypothetically, the local application may require a smaller dose to affect the targeted tissue compared to the systemic route by potentially decreasing the side effects of steroids. In the current study, we compared the therapeutic efficacy of LSI, including triamcinolone acetonide, with combined LSI and low-dose oral methylprednisolone in terms of response to therapy, outcome, and side effect profile, along with topical application of triamcinolone acetonide-containing pomade in patients with severe IGM. To our knowledge, this is the first study to compare LSI alone to LSI with low-dose steroid therapy.

There are different attitudes regarding the systemic use of steroids in the published literature. In 1980, De Herthogh et al. first reported a high-dose corticosteroid regimen starting with prednisolone 30 mg/day (9). However, patients suffer from a variety of side effects, such as weight gain, hyperglycemia, and cushingoid face. Freeman et al. suggested a low-dose regimen of 16 mg prednisolone twice a day by slow tapering over 2 months, with a similar side-effect profile (28). In a retrospective multicenter Turkish study of 720 patients by Uysal et al., the recurrence rate was 17% in patients with IGM that was not affected by different treatment modalities (29).

Considering the undesirable side effects of high-dose steroid therapy, we investigated the efficacy of LSI alone vs. that of LSI combined with low-dose OST. Briefly, the majority of patients (92.2%) showed either a complete or partial response in the present study. Furthermore, similar complete and partial response rates were observed in both cohorts following therapy completion. However, patients treated with LSI alone required more LSI applications than those in the combined therapy group to achieve an optimum response to therapy (LSI alone, 5 vs. combined group, 3.5; *p* = 0.08), which did not reach statistical significance. With this treatment strategy, the CR rate was 52% in the LSI group vs. 53.9% in the OST group in our series by physical examination and radiological evaluation, including ultrasonography and/or MRI, respectively. Furthermore, no significant difference was found in the 1-year recurrence rates between the LSI alone (4%) and the combined group (15.4%). In concordance with our report, Alper et al. reported that the total response and recurrence rates were 89.3 and 10.7%, respectively, in 28 patients with IGM treated with LSI alone (20). In another prospective cohort study by Alper et al. (21), LSI (*n* = 42) was compared to systemic oral treatment (*n* = 16). All patients were treated until a therapeutic response was obtained, similar to our study design. In concordance with our findings, the median treatment duration was 5 months in the local injection group and 3 months in the systemic group. No significant difference was found in the 2-year recurrence rates between the LSI group (4.8%) and systemic steroid treatment group (12.5%). Similarly, 70 of 72 IGM lesions (97%) responded completely to treatment in patients (*n* = 38) treated with LSI alone in another report by Ertürk et al. (23). More than half (22/38, 57.9%) of the patients required at least three LSI applications to obtain a satisfactory clinical response, with a complete clinical recovery of 94.5% after completion of the therapy. Furthermore, the number of applications needed for the full recovery of the patients differed between one and five applications according to the IGM severity based on the number and size of granulomas and the presence of abscess or fistula.

Similar to our study, Yildirim et al. compared the efficacy of intralesional (*n* = 17) and systemic steroid (*n* = 19) administration for the treatment of IGM in a prospective randomized controlled study (22). Patients treated with intralesional steroids required monthly multiple LSIs differing between 1 and 6 applications until the maximum clinical regression was achieved with the therapy as assessed by physical examination and imaging. The CR and recurrence rates were 88.2% (15/17) and 23.5 (2/17) in the LSI group and 78.9% (15/19) and 26.3% (4/19) in the OST group, respectively. No significant difference could be found in the CR and recurrence rates between the 2 groups in concordance with our study and other published reports that has a similar study design comparing LSI with systemic peroral steroid use in IGM (19, 21).

Finally, Toktas et al. (19) investigated the efficacy of LSI (*n* = 46) compared with systemic therapy (*n* = 32), and reported a better response and a lower recurrence rate in the LSI group (93.5 vs. 71.9%, *p* = 0.012; and 8.7 vs. 46.9%, *p* = 0.001), respectively. The need for surgical treatment was significantly higher in the systemic treatment group (9.4%) than in the LSI alone group (2.2%). In the present study, only one patient (3.8%) with localized IGM and partial response in the combined therapy group underwent surgery for residual disease despite multiple LSI applications with systemic treatment. The different response rates in the studies may be due to the different criteria used in the response assessment. Therefore, considering the total response rates, including complete and partial responses, might be a better strategy for comparing the efficacy of treatment between different protocols.

Intralesional steroid injections have recently evolved as a new therapeutic modality with promising results, with a potentially better side effect profile in patients with IGM (18–24). We also observed fewer steroid-related side effects in the LSI-alone group (32%) than in the combined group (69.3%), which is similar to the findings of recently published reports (19–22). Notably, patients with LSI alone were less likely to gain weight due to steroid therapy as the most remarkable decreased side effect of the LSI route in our study. Even though slight decreases were observed in other side effects, including skin thinning, menstrual irregularity, weakness, hirsutism, and facial edema, these differences were not statistically significant in our study. Furthermore, local side effects were also very few in the present cohort, as in only one patient (4%) with erythema in the injection area following the application, which resolved very rapidly in a few days, similar to the study by Toktaş et al. (19), who reported that local side effects were observed in one patient (2.2%) treated with steroid injections. However, systemic side effects were observed at a very low rate, as 9.4% of the patients received systemic treatment, which might be due to the relatively shorter duration of systemic exposure to steroids for a maximum of 3 months in their study. Ertürk et al. further reported that 2 of 38 patients developed hematoma after LSI (23). However, patients in other reports and in the present study were found to have a higher rate of steroid-related systemic side effects, even though patients in our study were treated with a low-dose peroral steroid regimen for a maximum period of >6 months in a few cases in the systemically treated group. These differences among studies might be due to the different criteria in the assessment of side effect profiles since we included mild side effects as well as moderate or severe side effects. Similar to Toktaş et al., Yildirim et al. (22) found no difference in the side effect profile between patients with LSI (10.5%) and the systemic steroid group (11.8%) reporting relatively lower complication rates compared to our study. However, Alper et al. reported higher rates of steroid-related side effects (14.3% in the local treatment group and 81.3% in the systemic treatment group), in concordance with our study demonstrating remarkably fewer side effects in patients with LSI than in the systemic treatment group (*p* < 0.001) (21).

Among the studies that applied LSI in patients with IGM, either 40 mg methylprednisolone (18, 20, 21) or 20 mg to 40 mg triamcinolone acetonide (19, 22–24) were used as the active ingredients. LSI was mostly applied under USG guidance in reported series (18–23). However, similar to the recent study by Ren et al. (24), we applied LSI to palpable granulomas without any USG guidance and obtained similar response rates in concordance with previously published reports (18–23). In the report of Ren et al., the authors have compared the therapeutic efficacy of systemic oral steroids alone starting at a dose (0.5 mg/kg) compared to the systemic oral steroids starting at a lower dose (0.25 mg/kg) combined with intralesional steroid injection (weekly 20 mg triamcinolone acetonide, maximum 4 times) in the preoperative setting. They showed a better therapeutic response of the combined therapy (group A) than oral steroids treatment alone (group B). They have observed a significant reduction in the granuloma size in group A (52.06%) vs. in group B (30.00%) with a significantly increased post-operative aesthetic satisfaction in group A. Furthermore, adding weekly intralesional steroid injections reduced the duration of oral steroid use from 7 weeks in group B to 4 weeks in group A (*p* < 001). However, no difference could be found between the two groups regarding the adverse effects and post-operative recurrence rate. Of note, all patients underwent surgery following steroid treatment either as oral treatment alone or combined with LSI which differs from our study along with other published studies as study design. Therefore, our study design in the present report seems to be unique by far which compares LSI-alone with the combined LSI and low-dose oral steroid therapy as the sole treatment modality without performing surgery.

## Conclusion

Local steroid injection could be considered as the first-line treatment in patients with severe IGM until a therapeutic response has been obtained either as the sole treatment modality or combined with oral steroids. We report here that the majority of patients with LSI, either alone or in combination with OST, achieved a complete or partial clinical response with a tolerable side effect profile. Patients in both groups showed similar response patterns to therapy. However, the duration of LSI therapy was longer in the LSI alone group, with fewer side effects. Therefore, compared with systemic oral steroid therapy, local steroid administration alone can be considered a new evolving treatment modality with fewer side effects. Prospective multicenter randomized trials with a higher number of patients that were allocated without bias and different designs based on the previous findings of the publications are to be studied to improve this new modality in the management of IGM.

## Data availability statement

The raw data supporting the conclusions of this article will be made available by the authors, without undue reservation.

## Ethics statement

The studies involving humans were approved by the Institute’s Ethical Committee of Istanbul University, Istanbul Faculty of Medicine (No: 1531637). The studies were conducted in accordance with the local legislation and institutional requirements. The participants provided their written informed consent to participate in this study. Written informed consent was obtained from the individual(s) for the publication of any potentially identifiable images or data included in this article.

## Author contributions

NC and CU designed the study. NC and HM performed the initial search, literature organization, analyses, and manuscript writing. NC, HM, DS, SE, and RY performed the data acquisition. SE, MT, OD, AA, HK, and MM made critical comments and typesetting corrections to the final version. All authors have read and revised the manuscript.
